# Leptin and leptin receptor polymorphisms are associated with increased risk and poor prognosis of breast carcinoma

**DOI:** 10.1186/1471-2407-6-38

**Published:** 2006-02-20

**Authors:** Kaouther Snoussi, A Donny Strosberg, Noureddine Bouaouina, Slim Ben Ahmed, A Noureddine Helal, Lotfi Chouchane

**Affiliations:** 1 Laboratoire d'Immuno-Oncologie Moléculaire, Faculté de Médecine de Monastir, Université de Monastir, Tunisia; 2 Department of Infectology, Scripps-Florida, USA; 3 Department of Cancérologie Radiothérapie CHU Farhat Hached, Sousse, Tunisia; 4 Department of Service de Carcinologie Médicale, CHU Farhat Hached, Sousse, Tunisia; 5 Unité Génome, Diagnostic Immunitaire et Valorisation, Institut Supérieur de Biotechnologie de Monastir, Université de Monastir, Tunisia

## Abstract

**Background:**

Leptin (**LEP**) has been consistently associated with angiogenesis and tumor growth. Leptin exerts its physiological action through its specific receptor (**LEPR**). We have investigated whether genetic variations in ***LEP ***and ***LEPR ***have implications for susceptibility to and prognosis in breast carcinoma.

**Methods:**

We used the polymerase chain reaction and restriction enzyme digestion to characterize the variation of the ***LEP ***and ***LEPR ***genes in 308 unrelated Tunisian patients with breast carcinoma and 222 healthy control subjects. Associations of the clinicopathologic parameters and these genetic markers with the rates of the breast carcinoma-specific overall survival (OVS) and the disease free survival (DFS) were assessed using univariate and multivariate analyses.

**Results:**

A significantly increased risk of breast carcinoma was associated with heterozygous ***LEP (-2548) GA ***(OR = 1.45; *P *= 0.04) and homozygous ***LEP (-2548) AA ***(OR = 3.17; *P *= 0.001) variants. A highly significant association was found between the heterozygous ***LEPR 223QR ***genotype (OR = 1.68; *P *= 0.007) or homozygous ***LEPR 223RR ***genotype (OR = 2.26; *P *= 0.001) and breast carcinoma.

Moreover, the presence of the ***LEP (-2548) A ***allele showed a significant association with decreased disease-free survival in breast carcinoma patients, and the presence of the ***LEPR 223R ***allele showed a significant association with decreased overall survival.

**Conclusion:**

Our results indicated that the polymorphisms in ***LEP ***and ***LEPR ***genes are associated with increased breast cancer risk as well as disease progress, supporting our hypothesis for leptin involvement in cancer pathogenesis.

## Background

Breast cancer is the most frequently diagnosed cancer among women, with a lifetime incidence of about 10–13% [1,2] but its etiology is still not fully understood. Besides, age at menarche and menopause, diet, reproductive history, estrogen administration and genetic factors have been suggested as risk factor for breast cancer [3-7]. Germline mutations in dominant, highly penetrant susceptibility genes, such as BRCA1 and BRCA2, have been identified to be responsible for less than 10% of breast carcinoma. In addition, low-penetrance genes, that also increase susceptibility to breast cancer, have been suggested. For instance, candidate low-penetrance breast cancer susceptibility genes include those involved in the complex mechanisms of carcinogenesis [8].

Higher body weight appears to play a role in the development of breast/mammary tumors. Obesity has been identified as a risk factor for breast cancer in postmenopausal women [9-11], and higher body weight is associated with increased incidence of both spontaneous and chemically induced mammary tumors in rodents [12-15].

Leptin, a 16 kDa polypeptide hormone produced predominantly by white adipose tissue [16], plays an important role in body weight homeostasis through effects on food intake and energy expenditure [17,18]. In addition to the regulation of body weight, leptin also influences hematopoiesis, reproduction, angiogenesis, and immune processes [19-22]. The leptin gene, the human homologue of the rat obese gene (OB), has been cloned and sequenced by Zhang et al. [16]. It is located on chromosome 7q31.3 [23,24] and expresses a 4.5 kb mRNA in adipose tissue [16,25].

Leptin exerts its physiological action through the leptin receptor (LEPR), a member of the class 1 cytokine receptor family. LEPR was initially identified in the brain, which explains the negative feedback mechanism of controlling food intake and body weight [26]. However, further studies have demonstrated that LEPR is also expressed in many other tissues and cells, including placenta, pancreas, hematopoietic cells, liver, lung and gastric mucosal cells [27-32].

The human ***LEPR ***gene is on chromosome 1. Six isoforms derived from ***LEPR ***transcription have been identified, and a long isoform, LEPR-b, is reported to be responsible for signal transduction [33].

In animal and human cell lines, leptin and leptin receptors have also been clearly associated with enhanced *in vitro *tumor proliferation and/or to *in vitro *and *in vivo *promotion of angiogenesis. These effects have been documented in embryonic cells [34], adipocytes [35], glia [36], endothelial cells [37], hematopoietic cells [38], and in benign and malignant epithelial breast cells [39-42], kidney, colon [43,44], liver [45], and pancreas [46].

In breast cancer tissue, it was shown that leptin and leptin receptor are both expressed and that they act to favour cancer proliferation and metastasis [47,48]. Controversial results have been reported regarding the detection of serum leptin levels in breast cancer patients [49,50]. However, the most recent reports indicate that higher leptin serum levels are associated with advanced stage breast cancer [51,52].

In humans, several polymorphisms have been identified in the ***LEP ***and ***LEPR ***genes: a ***G ***to ***A ***substitution at nt -2548 upstream of the ATG start site [53] in the ***LEP ***gene 5' promoter region, and an ***A ***to ***G ***substitution at nt 668 from the start codon 223 in exon 6 (Q223R) of the ***LEPR ***gene coding for the extracellular region common to all isoforms of ***LEPR ***[54].

The ***G ***to ***A ***substitution at -2548 of the ***LEP ***gene was associated with leptin production [55,56]. The glutamine to arginine substitution occurs on the ***LEPR ***gene within the first of two putative leptin-binding regions and may be associated with impaired signalling capacity of the leptin receptor [57].

Enhanced gene expression and increased circulating leptin levels have been reported in subjects carrying the ***LEPR 223R ***or ***LEP (-2548) A ***alleles [55-57]. The ***LEP (-2548) A ***allele has also been associated with a two-fold increase of leptin secretion adipocytes when compared to secretion by adipocytes bearing only the ***LEP (-2548) G ***allele [56].

We hypothesize that these leptin and leptin receptor polymorphisms associated with higher leptin serum levels and overexpression of leptin in adipocytes, favour breast cancer development and aggressiveness. In the present study, we analysed ***LEP (-2548) G/A ***and ***LEPR Q223R ***genotypes in a series of breast cancer cases and normal controls.

## Methods

### Patients and controls

The gene and allele frequencies of the ***LEP ***and ***LEPR ***genes were determined in a group of 222 control subjects and 308 patients with breast carcinoma. Controls and patients were selected from the same population living in the middle coast of Tunisia. Both the control and patients groups include unrelated subjects.

Data on patient, tumour and treatment characteristics at the study entry for each subject were collected from the department of Radiation Oncology and Medical Oncology of Sousse Hospital (Sousse, Tunisia) between 1996 and 2003. They were selected consecutively whenever practically feasible.

All patients included in this study had primary breast carcinoma, with unilateral breast tumours and with no family history for the disease. The diagnosis of cancer was confirmed by histopathology analyses. The patients (n = 308) had a mean age of 50 ± 24 years. The median of follow-up was 36 months (range, 1–120 months). At time of analysis, 66 patients experienced recurrence (local or distant). Among them, 35 died from breast carcinoma (54.5 %). Table 1 shows the treatment description of all patients. A detailed description of the clinicopathologic characteristic of this cohort has been reported elsewhere [58] and data on tumor size at diagnosis, nodal status and histologic grade are briefly included in Table 3.

Healthy women (n = 222) having a mean age of 48 ± 14 years, were blood donors with no evidence of any personal or family history of cancer (or other serious illness). Samples from healthy controls were collected consecutively between 1996 and 2003. Both cases and controls were informed and gave written consent to participate in the study and to allow their biological samples to be genetically analyzed. Approval for the study was given by the National Ethical Committee.

### Patients treated by chemotherapy as a primary treatment

Among the 308 patients, 121 had chemotherapy as an anticancer primary treatment. The chemotherapy induction was based in all cases on the combination of cyclophosphamide (100%), 5-fluorouracil (100%) with adriamycin (34%) or epirubicin (52%) or methotrexate (14%). For neoadjuvant treatment, patients received four or six chemotherapy cycles before surgery. The clinical response to induction chemotherapy for all cases was defined according to the following criteria: complete response when regression of the tumour was total, partial response when reduction of tumour size was greater then 50% and poor response when the reduction of tumour size was less then 50% (tumour size was measured by the bidimensional product of the horizontal and vertical dimensions).

### Genomic DNA extraction

Genomic DNA was extracted from peripheral blood leukocytes by a salting procedure [59]. Briefly, 10 ml of blood was mixed with triton lysis buffer (0.32M sucrose, 1% Triton X-100, 5 mM MgCl_2_, H_2_O, 10 mM Tris-HCl, PH 7.5). Leukocytes were spun down and washed with H_2_O. The pellet was incubated with proteinase K at 56°C and subsequently salted out at 4°C using a substrate NaCl solution. Precipated proteins were removed by centrifugation. The DNA in supernatant fluid was precipated with ethanol. The DNA pellet was dissolved in 400 μl H_2_O.

### Polymorphism analysis of the -2548 G/A LEP gene

The ***-2548 G/A ***polymorphism of the ***LEP ***gene was analysed by restriction fragment length polymorphism-polymerase chain reaction (RFLP-PCR). This method was carried out by PCR amplification using forward primer 5'-TTTCTGTAATTTTCCCGTGAG-3' and reverse primer 5'-AAAGCAAAGACAGGCATAAAAA-3' in a 25 μl reaction mixture containing genomic DNA samples (100 ng), 200 μmol/L dNTPs, 1.5 mM MgCl_2_, 1 X Taq polymerase buffer, 50 pmol of each primer, and 0.5 unit of Taq DNA polymerase (Amersham, Paris, France). Reaction conditions used with the thermal cycler (Biometra, GÖttingem, Germany) were as follows: an initial incubation at 94°C for 5 minutes followed by 30 cycles of incubation at 94°C for 45 seconds, 52°C for 45 seconds and 72°C for 45 seconds with a final extension at 72°C for 7 minutes. The PCR product (242 bp) was verified by DNA sequencing.

The amplified products were digested with the addition of 2 U *Cfo*I for 3 hours at 37°C. The digested samples were separated by electrophoresis through a 2% agarose gels and stained with ethidium bromide. The polymorphism was defined by presence (***G***) or absence (***A***) of the *Cfo*I restriction site. To assess reliability of genotyping we performed double sampling RFLP-PCR in more than 10% of the samples and found no differences.

### Polymorphism analysis of the LEPR gene

Based on the method described by Gotoda et al. [54], a PCR followed by digestion with endonuclease *Msp*I was used to detect the ***A ***to ***G ***transition polymorphism at codon 223 of ***LEPR ***gene. Two sequence specific oligonucleotides primers were used for the polymerase chain reaction (PCR): the 3' primer 5'-AAACTCAACGACACTCTCCTT- 3' and 5' primer 5'-TGAACTGACATTAGAGGTGAC- 3'. PCR was performed by using 100 ng genomic DNA as template, 0.01 mM dNTPs, 1.5 mM MgCl_2_, 1 X Taq polymerase buffer, 0.75μM of each primer and 0.5 U of Taq DNA polymerase in a total reaction of 25 μl. Reaction conditions used with the thermal cycler were as follows: 94°C for 5 min followed by 35 cycles at 94°C for 30 seconds, 52°C for 45 seconds and 72°C for 45 seconds. A final extension step was carried out at 72°C for 5 min.

The PCR products (80 bp) were electrophoresed on a 3% agarose gel containing ethidium bromide to monitor amplification and possible contamination. The PCR product (80 bp) was verified by DNA sequencing. The 80 bp PCR products were digested with *Msp*I and analysed on 4% (3:1 Nusieve: Normal) agarose gels because of the small DNA products size. The presence of *Msp*I site was indicated by the cleavage of the amplified product into two fragments of 57 and 23 bp. The two allelic forms of ***LEPR***, corresponding to the absence or the presence of the *Msp*I site, are referred to as ***223Q ***and ***223R***, respectively.

### Statistical analyses

The allele frequencies of ***LEP ***and ***LEPR ***were tested for the Hardy-Weinberg equilibrium for both patient and control groups using the chi-square test. The same test was used to evaluate for significant association between disease (breast carcinoma *versus *controls) and ***LEP ***or ***LEPR ***genotypes or alleles.

Disease-free survival (DFS) was defined as the time from the date of diagnosis to the first local or distant recurrence or to last contact. Breast carcinoma-specific overall survival (OVS) was defined as the time from the date of diagnosis to death if the patient died from breast carcinoma or to last contact. Six-year survival rates were estimated, and survival curves were plotted according to Kaplan and Meier [60]. The differences between groups were calculated by the log-rank test [61].

Clinicopathological parameters were dichotomised as follows: nodal status (≥ 1 *versus *no positive lymph node), SBR (Scarff, Bloom and Richardson) tumour grade (1–2 *versus *3), clinical tumour size (T_1_-T_2 _*versus *T_3_-T_4_).

Statistics were performed using SEM-STATISTIQUES software (centre Jean Perrin, Clermont-Ferrand, France).

## Results

### Polymorphisms in the LEP and LEPR genes as risk factor for breast carcinoma

The genotype distribution and allele frequencies for the ***LEP (-2548) G/A ***and ***LEPR Q223R ***polymorphisms in all patients with breast carcinoma and in the control group are presented in Table 2. The allele frequencies of ***LEP ***and ***LEPR ***genes were in Hardy-Weinberg equilibrium in both patient and control groups (*P *= 0.55, *P *= 0.7; *P *= 0.17, *P *= 0.376 respectively).

A significantly higher risk for breast cancer was observed for carriers of ***LEP (-2548) AA ***genotype (Odds Ratio (OR) = 3.17; *P *= 0.001) and carriers of ***LEP (-2548) GA ***genotype (OR = 1.45; *P *= 0.04). The ***LEP (-2548) A ***allele frequency was significantly higher in the patient group compared to the control group (OR = 1.55; *P = *0.001).

The allelic frequency of the ***LEPR 223R ***was 0.446 in patients with breast carcinoma and 0.338 in control subjects (OR = 1.57; *P *= 0.0003). The frequency of heterozygous ***LEPR 223QR ***was 0.471 in the patient group and 0.405 in the control population resulting in a significantly increased OR in the patient group bearing this genotype (OR = 1.68; *P *= 0.007). The frequency of the homozygous ***LEPR 223 RR ***was 0.211 in the patient group and only 0.135 in controls (OR = 2.26; *P *= 0.001). These data, taken together, suggest that an increased risk of developing breast carcinoma is associated with the inheritance of the ***LEP (-2548) A ***and ***LEPR 223R ***alleles in a dose-dependant manner.

When we stratified the patients according to the menopause status (190 pre-menopausal and 118 post-menopausal) no significant changes in the ***LEP ***and ***LEPR ***genotype distributions were seen in breast carcinoma subgroups (data not shown).

### Prognostic significance of polymorphism in LEP and LEPR genes

Table 3 shows the distributions of ***LEP ***and ***LEPR ***polymorphisms according to the clinic-pathologic indices of breast carcinoma severity.

We stratified patients according to tumor size in two subgroups. The first group includes tumor size less than 5 cm (T1-T2) and the second group includes patients with locally advanced breast cancer (T3-T4). The frequency of the ***LEP (-2548) A ***allele was significantly higher in patients with larger tumor size (*P *= 0.05). No association was found with the other clinic-pathologic parameters.

When we tested the relationship between the presence of the ***LEP (-2548) A ***allele in all the 308 patients and the survival (OVS and DFS), significant differences were observed between the DFS Kaplan-Meier survival curves for the different polymorphisms.

**Figure 1:**
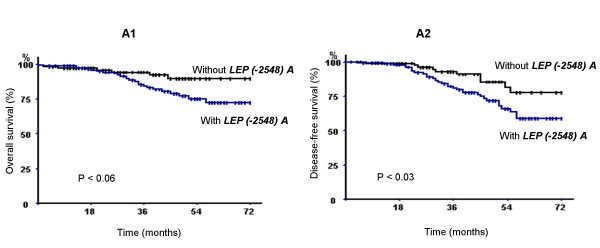
Breast carcinoma specific overall survival (A1) and specific disease-free survival (A2) of 308 breast carcinoma patients according to the presence or absence of ***LEP (-2548) A ***allele (*p *denotes the log-rank test value).

As shown in figure 1, the breast carcinoma specific DFS was significantly shorter in the patient population carrying the ***LEP (-2548) A ***allele. The estimated 3- and 6- year breast carcinoma specific DFS rates in the group of patients carrying the ***LEP (-2548) A ***allele were, respectively, 85.29 % and 60.29 % versus the DFS rates of 95.59 % and 79.41 % for those carrying the ***LEP (-2548) G ***allele (log rank test, *P *< 0.03). No statistical difference in overall survival between both groups of patients was observed.

**Figure 2:**
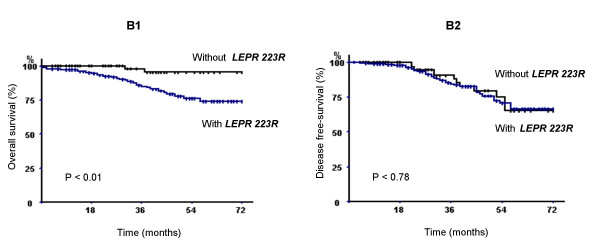
Breast carcinoma specific overall survival (B1) and specific disease-free survival (B2) of 308 breast carcinoma patients according to the presence or absence of ***LEPR 223R ***allele (*p *denotes the log-rank test value).

The breast carcinoma specific OVS was significantly shorter in the group of patients carrying ***LEPR 223R ***allele (Fig. 2). The estimated 3- and 6- year breast carcinoma OVS rate in the group of patients carrying ***LEPR 223R ***allele were, respectively, 86.76 % and 75 % versus 98.5 and 97.06% for those carrying the ***223Q ***allele (log rank test, *P *< 0.01). In contrast, no association was found between ***LEPR 223R ***and the DFS rate in this population of patients with breast carcinoma.

Multivariate analyses were undertaken to evaluate the importance of ***LEP ***and ***LEPR ***markers in the risk of the recurrence and death compared with the clinicopathologic parameters. Introducing the genetic and the clinicopathologic parameters bearing prognostic significance has tested the Cox model. No genetic and clinicopathologic parameters were selected for OVS and DFS.

## Discussion

Leptin is a cytokine produced mainly by adipose tissue. Leptin has recently been reported to stimulate the proliferation of various cell types and is considered to be a new growth factor. Hyperleptinemia is a common feature of obese women who have a risk of breast cancer higher than those with normal weight and epidemiologic studies have suggested a positive correlation between obesity and breast cancer risk [62].

In addition, recent studies indicate that leptin is a mitogenic [63], as well as pro-angiogenic factor in various cell models. New data documented that human breast cancer cell lines and breast tumors may express leptin and leptin receptors [40,42,47,48]. These characteristics of leptin prompted us to evaluate whether genetic variations of the ***LEP ***gene promoter and/or ***LEPR ***exon 6 affect susceptibility to and prognosis of breast cancer.

The present case/control study showed a substantially increased risk of breast carcinoma associated in a dose-dependent manner, with the inheritance of the ***LEP (-2548) A ***allele. Individuals homozygous for ***LEP (-2548) A ***have more than 3-fold risk to develop breast carcinoma (OR = 3.17; *P *= 0.001) compared with individuals homozygous for ***LEP (-2548) G ***allele. The ***LEP (-2548) GA ***heterozygotes patients have an intermediate risk for this cancer (OR = 1.45; *P *= 0.04).

Comparison of genotype frequencies of the ***LEPR Q223R ***alleles in patients with breast carcinoma and control subjects indicated an increase of ***LEPR 223QR ***and ***RR ***genotypes. Consequently, the frequency of the ***LEPR 223R ***allele was found to be significantly higher in patients group compared with controls.

In this study, we investigate the prognostic significance evaluation of ***LEP ***and ***LEPR ***polymorphism by examining the potential association of ***LEP ***-2548 and ***LEPR ***exon 6 variations and the clinical response to chemotherapy induction. This evaluation indicated that ***LEP ***and ***LEPR ***polymorphisms do not have a predictive value for clinical responses to chemotherapy.

The prognostic evaluation of the ***LEP ***and ***LEPR ***genetic markers in breast carcinoma indicated that ***LEP (-2548) A ***allele is associated with a larger tumor size at diagnosis and a shorter disease-free survival, and that the ***LEPR 223R ***allele is associated with shorter overall survival, and therefore with a poor prognosis in breast carcinoma.

Both functional studies and genetic analyses have highlighted the role of LEP and LEPR in cancer pathogenesis and disease progression [64-67].

Previous reports demonstrated that ***LEP (-2548) A ***allele was significantly associated with higher leptin level [55,56]. However, the relationship between leptin level and the ***LEPR Q223R ***polymorphism are conflicting. Quinton et al. have indicated that ***LEPR 223R ***allele was associated with lower circulating leptin level [68]. In contrast, Yiannakouris et al. have found that carriers of the ***LEPR 223R ***allele had significantly higher leptin level than non carriers [57].

Several studies have shown the relationship between ***LEP ***and ***LEPR ***gene polymorphisms and human cancer [66,67,69]. The ***LEP (-2548) A ***allele, which result in high leptin secretion, was associated with increased risk of prostate cancer [66]. Recently, a study conducted on 45 patients has reported that ***LEPR Q223R ***polymorphism is not associated with an increased risk of breast cancer [69]. This result is in contrast with our findings.

The relationship between leptin serum level and breast cancer is still controversial. Mantzoros et al. were observed that leptin did not appear to increase the risk of pre-menopausal breast cancer in situ [70]. In addition, Petridou et al. have found no relationship between leptin serum levels and breast cancer in post-menopausal women [49]. A recent large study has demonstrated that subjects with elevated serum leptin levels displayed increased risk of developing breast cancer than those with the normal levels [52]. Furthermore, in the same study, the serum level of leptin was not associated with menopausal status in patients with mammary disease. This result along with our findings suggests that leptin increased the risk of breast cancer independently from menopausal status.

Additionnaly, in breast cancer it has been shown that a high leptin serum level is associated with reduced OVS [71]. Recently, it has been reported that the levels of expression of leptin and leptin receptor correlate with distant metastasis [47]. Patients with LEPR negative and low leptin-expressing tumors have shown an extremely good outcome, and the survival rate tended to be lower for patients with LEPR positive or high leptin-expressing tumors [47].

Some authors have demonstrated that leptin increase cell proliferation which is an essential element for tumor metastasis, through cell progression in MCF-7 human breast cancer cells with up-regulation of PKC-α, PPARγ and PPARα [41]. Moreover, Laud et al. have suggested that leptin stimulated the proliferation of the mitogen-activated protein kinase (MAP kinase) pathway [40]. Leptin may also modulate apoptosis, since it has been reported in leukaemia cell lines that leptin acts as an inhibitor of apoptosis [72]. In addition, leptin has been shown to promote invasiveness of renal and colonic epithelial cells via the phosphatidylinositol 3'-kinase, rho-dependent, and rac-dependent cascades [43]. Others have also reported that leptin act synergistically with VEGF and fibroblast growth factor 2 (FGF-2) to promote angiogenesis [73,74]. Moreover, leptin has been shown to increase the expression of many other genes involved in angiogenesis, for example, MMP-2 and MMP-9 gene products [75].

Other lines of evidence derive from studies on markers of tumoral risk that are elevated in breast cancer, that can upregulate leptin production. Factors such as TNF-alpha [76,77], IL-1alpha [78], IL-1 beta [79], estrogens [79], all raise leptin levels, promoting growth and differentiation of tumors.

The results of the current study, which show the association between the ***LEP (-2548) G/A ***and ***LEPR Q223R ***polymorphisms and breast carcinoma susceptibility and survival, suggest that the genetic basis of the potential tumor promoter role attributed to ***LEP ***and its receptor may result from certain ***LEP ***and ***LEPR ***polymorphisms. Therefore, these polymorphisms may play a casual role in tumor development as well as in governing poor prognosis of breast carcinoma.

## Conclusion

In conclusion, this study suggests that genetic variation in the tandem ***LEP ***and its receptor ***LEPR ***may be attractive susceptibility markers for breast carcinoma. These genetic markers represent also, prognostic variables for predicting recurrence and death from breast carcinoma.

The role of the leptin and its receptor as genetic markers for breast cancer can be completed with other SNPs and a haplotype analysis.

## Competing interests

The author(s) declare that they have no competing interests.

## Authors' contributions

SK conceived of the study, conducted data analysis, and drafted the manuscript. AD-S contributed to the design and management of data. NB and S-BA provided samples and clinical information. LC conceived, designed and participated in the data analysis and interpretation of the study. AN-H contributed to reviewing the manuscript. All authors read and approved the final manuscript.

## Pre-publication history

The pre-publication history for this paper can be accessed here:



## Figures and Tables

**Table 1:** Treatment description of the 308 patients with Breast Carcinoma

	**Surgery**	**No surgery**
Radiotherapy	33	14
Chemotherapy	28	14
Radiotherapy + Chemotherapy	137	18
Radiotherapy + endocrine therapy	14	0
Chemotherapy + endocrine therapy	7	4
Radiotherapy + Chemotherapy + endocrine therapy	39	0

**Table 2:** The *LEP *(-2548) *G/A *and *LEPR Q223R *Genotype distributions in Control Subjects and in Patients with Breast Carcinoma.

**Genotypes**	**Patients (n = 308)**		**Controls (n = 222)**		**OR**	**Confidence interval**	***P*-value**
	*n*	*f*	*n*	*f*			
***LEP (*-2548) *G/A***							
GG	119	(0.386)	112	(0.504)	1		
GA	152	(0.494)	99	(0.446)	1.45	[0.99–2.11]	0.04
AA	37	(0.120)	11	(0.050)	3.17	[1.47–6.96]	0.001
**Alleles**							
G-allele	390	(0.633)	323	(0.727)			
A-allele	226	(0.367)	121	(0.273)	1.55	[1.18–2.04]	0.001
							
***LEPR Q223R***							
QQ	98	(0.318)	102	(0.460)	1		
QR	145	(0.471)	90	(0.405)	1.68	[1.12–2.50]	0.007
RR	65	(0.211)	30	(0.135)	2.26	[1.31–3.90]	0.001
**Alleles**							
Q-allele	341	(0.554)	294	(0.662)			
R-allele	275	(0.446)	150	(0.338)	158	[1.22–2.05]	0.0003

OR, odds ratio; CI, confidence interval. The χ^2 ^test was used to determine whether significant differences (*P*-value) were observed when the patient group was compared with the control group. *f*, frequencies.

**Table 3:** Genotype frequencies of *LEP *and *LEPR *polymorphisms in relation to pathological indices of breast cancer severity.

Pathological feature		Number of patients (%)	P*-value*
***LEP *(-2548) G/A**	**GG**	**GA + AA**	
			
**Tumor size**			
T1-T2	82 (42.05)	113 (57.95)	**0.05**
T3-T4	21 (26.92)	57 (73.08)	
**Histological grade**			
1–2	68 (39.08)	106 (60.92)	0.3
3	28 (32.56)	58 (67.44)	
**Lymph node metastases**			
Negative	64 (42.38)	87 (57.62)	0.13
Positive	51 (34.00)	99 (66.00)	
**Hormone receptor status**			
Negative	34 (38.20)	55 (61.80)	0.36
Positive	77 (44.00)	98 (56.00)	
			
***LEPR Q223R***	***QQ***	***QQ + RR***	
**Tumor size**			
T1-T2	56 (29.17)	136 (70.83)	0.57
T3-T4	27 (32.53)	56 (67.47)	
**Histological grade**			
1–2	58 (32.95)	118 (67.05)	0.19
3	21 (25.00)	63 (75.00)	
**Lymph node metastases**			
Negative	55 (36.67)	95 (63.33)	0.07
Positive	41 (27.15)	110 (72.85)	
**Hormone receptor status**			
Negative	40 (41.53)	49 (58.47)	0.57
Positive	85 (34.33)	90 (65.67)	

OR, odds ratio; CI, confidence interval. The χ^2 ^test was used to determine whether significant differences (*P*-value) were observed when the patient group was compared with the control group. *f*, frequencies.
